# Infrared Thermal Imaging System on a Mobile Phone

**DOI:** 10.3390/s150510166

**Published:** 2015-04-30

**Authors:** Fu-Feng Lee, Feng Chen, Jing Liu

**Affiliations:** 1 Department of Biomedical Engineering, School of Medicine, Tsinghua University, Beijing 100084, China; E-Mail: ffleefflee@gmail.com; 2 Department of Automation, Tsinghua University, Beijing 100084, China; E-Mail: chenfeng@mail.tsinghua.edu.cn

**Keywords:** infrared thermal imaging system, mobile phone, non-uniformity corrections, sudden infant death syndrome, pervasive technology, wireless monitoring

## Abstract

A novel concept towards pervasively available low-cost infrared thermal imaging system lunched on a mobile phone (MTIS) was proposed and demonstrated in this article. Through digestion on the evolutional development of milestone technologies in the area, it can be found that the portable and low-cost design would become the main stream of thermal imager for civilian purposes. As a representative trial towards this important goal, a MTIS consisting of a thermal infrared module (TIM) and mobile phone with embedded exclusive software (IRAPP) was presented. The basic strategy for the TIM construction is illustrated, including sensor adoption and optical specification. The user-oriented software was developed in the Android environment by considering its popularity and expandability. Computational algorithms with non-uniformity correction and scene-change detection are established to optimize the imaging quality and efficiency of TIM. The performance experiments and analysis indicated that the currently available detective distance for the MTIS is about 29 m. Furthermore, some family-targeted utilization enabled by MTIS was also outlined, such as sudden infant death syndrome (SIDS) prevention, *etc.* This work suggests a ubiquitous way of significantly extending thermal infrared image into rather wide areas especially health care in the coming time.

## 1. Introduction

Along with the growth of modern science, the thermal infrared imaging system is quickly emerging as an ever-powerful solution with diverse capabilities to detect temperature. Such measurements were often performed using thermometers, thermocouples, and resistance temperature detectors (RTD). Such instruments, however, can only obtain temperatures at specific points and need contact with the target. An alternative, the thermal imager, converts the infrared radiation emitted from the target into digital data and thus visualizes the whole temperature map through the infrared focal plane array (IRFPA) device. Therefore, the thermal imager allows for an outstanding nondestructive temperature measurement in many complex situations. This feature makes the utilization of such devices rather competitive with quite a few other methods, which promises its profound impact in many coming practices.

Despite the above-mentioned advantages in temperature measurement, a series of technical barriers still impede the wide adoption of thermal imager. Owing to the difficult fabrication of sensors, the thermal imager is usually high-priced, even for commercial purposes. For example, the current market price for a low-cost thermal imager, generally costs around 1000 US Dollars, which prevents the thermal imager from becoming easily accessible consumer electronics. Besides, most of the thermal imagers are lacking wireless communication ability and require cable or Secure Digital (SD) memory card to transfer data to the client. Although some thermal imagers provide wireless capability to the tablets and mobile phone, it seems that the client is not involved in the image processing and data calibration. Therefore, such machine serves as almost just like a graphical user interface or display unit. Apparently, the powerful computing capacity of the mobile phone is not fully used.

Aiming to develop a pervasively available thermal imager, here we demonstrated a novel concept of low-cost thermal imager launched on the mobile phone, the MTIS (mobile infrared image system). With the advantage of wireless capability and easy accessibility, the MTIS would provide a brand-new tool for future thermal image applications.

## 2. Digestion on Evolution of Thermal Imaging System

### 2.1. The Evolution History of Thermal Imager

**Figure 1:**
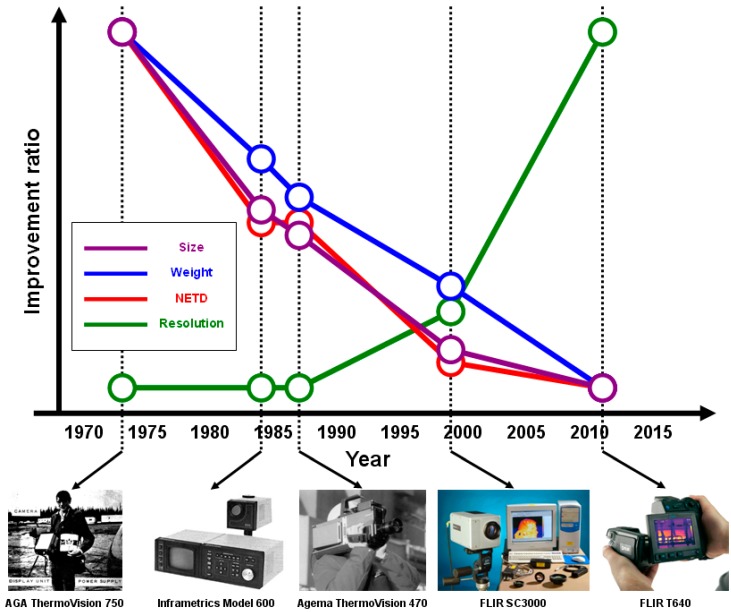
Relative improvement in some representative thermal imaging systems spanning from the year 1970 to 2015.

If performing a brief interpretation on the thermal imager’s evolution history, one can find that the innovations in this area were mainly dedicated to the realization of low cost, portability and minimization of the system (Figure 1). Earlier thermal imagers were generally separated into several different parts, including the power supply pack, the display unit, and the camera [1]. The first single-piece thermal imager, the Agema Thermovision 470, was eventually invented in 1987 [2]. Earlier thermal imager had to adopt a series of scanning mechanisms inside the camera for a single-point (or line) infrared detector to acquire a 2-dimensional image. Those complex mechanisms were gradually simplified or even eliminated after the advent of IRFPA.

Depending on the material type of the detector, additional strategy was necessary to implement inside the camera, and thus the size and weight of the camera was inevitably increased. For the photonic-type detector, a Dewar filled with liquid nitrogen or a Stirling Cycle cooler was added to cool down the detectors to a low temperature to reduce the thermal noise. For the pyroelectric-type detector, though it can operate at room temperature, a spinning slotted wheel (the so-called “chopper”) was added inside the camera to restore the sensitivity of the detector by interrupting the optical path between the focus lens and the detector [3]. Consequently, innovators were eager to find a new type of uncooled thermal infrared (UTIR) detector.

In the early 1990s, Honeywell, Inc. invented their own UTIR detector, the so-called “microbolometer” in today’s term. They chose the vanadium dioxide thin films—a kind of thermoresistive-type material—as the infrared absorbing medium, so the detector did not require cooling any more. Furthermore, benefiting from the material properties, the adoption of vanadium dioxide is quite compatible with the Complementary Metal-Oxide-Semiconductor (CMOS) process technology [4]. Such compatibility makes the microbolometer a valuable UTIR detector for modern thermal imager. Today, the one-hand-held design thermal imager has already been achieved.

### 2.2. Low-Cost Pursuit

As the thermal sensing technology becomes mature, more and more researchers started being involved in the development of low-cost thermal imagers. Although the microbolometer plays the leading role in the thermal imager, some innovators are still trying to find options based on the cost consideration. The main reason lies in that the know-how of micromachining, vacuum packaging, and materials preparation significantly raise the price of microbolometer. Since such issue seems hard to overcome in the near future, innovators are highly concerned about a more pervasive and cheaper UTIR detector—the thermopile.

Compared with the microbolometer, the thermopile has a big advantage in price and is easy to obtain from the market. With recent improvements in the resolution and sensitivity of thermopile IRFPA, the thermopile detector has been regarded as among the best cost-performance ratio solution for thermal imager development. Today, the price of low-cost thermal imagers based on thermopile detector is reduced to below 1000 US dollars. However, such price is still not easily acceptable when it comes to consumer electronics.

In order to further reduce the cost, an interesting idea that combines the thermal imager with a mobile phone is proposed in this work. The reason why we closely associate our work with mobile phone is that, if the mobile phone plays a role in the computing as well as display unit, the hardware cost of thermal imager can therefore be shared. The recent computing capacity of the mobile phone is potentially capable of running the thermal imaging program. More important, of course, is that the mobile phone, nowadays, has become a daily necessity for most people. Consumers’ opinion is that it is rather convenient and economical to buy a functional module combined with existing equipment. In this sense, the MTIS can be regarded as an ideal low-cost thermal imager for civilian purposes.

## 3. Experimental Setup

### 3.1. The System Overview of MTIS

**Figure 2:**
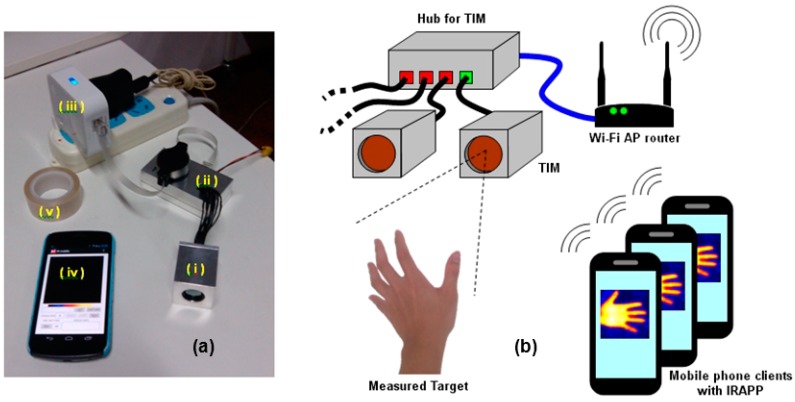
(**a**) The overview of MTIS testing console: (i) TIM; (ii) hub for TIM; (iii) Wi-Fi AP router; (iv) mobile phone client with IRAPP; (v) black body tape for accurate temperature measurement (optional); and (**b**) the architecture of MTIS testing console.

In this work, a measurement console that provides the essential function of thermal imaging and temperature measurement is developed. In the console, several thermal sensing modules (Thermal Infrared Module, TIM) are coordinated in a hub to locate different positions. The whole architecture is built on a wireless network with a Wi-Fi router, and the mobile phone plays the role of the console in the control unit (Figure 2). On the mobile phone, there is an exclusive software (Infrared Imaging Application software, IRAPP) to achieve the remote controlling and image display. When one of the mobile phone clients connects to the console, the IRAPP will show the IP address of the reacted TIM to indicate the monitoring position. Then, the TIM receives the thermal radiance from the measured target and reports the thermal data back to the client. The IRAPP handles the user datagram protocol (UDP) and data calculation, and finally displays the thermal image.

### 3.2. TIM Development

The TIM consists of a 64 × 62 thermopile IRFPA sensor (released by Heimann Sensor GmbH), a microcontroller, and Ethernet controller circuits. Previous research revealed that low-resolution thermal device with 64 × 64 pixels is adequate for home security and human recognition [5]. Therefore, such resolution of TIM is considered to fulfill the task in this study.

**Figure 3:**
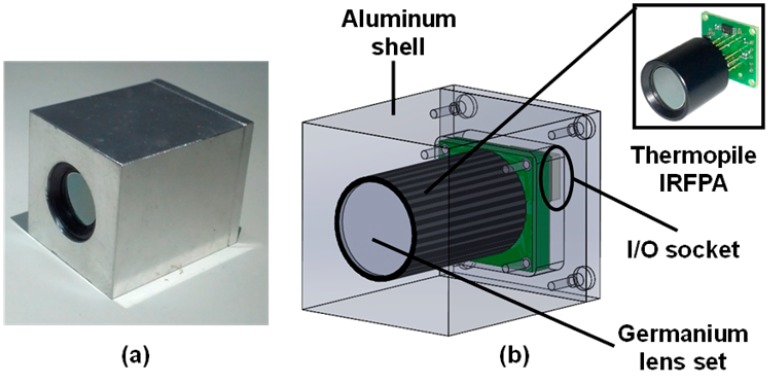
The design of Infrared Thermal Module (TIM). (**a**) Integrated TIM working prototype; (**b**) The interior structure of TIM.

As shown in Figure 3, the sensor is assembled with single germanium lens set to simplify the optical mechanism. The surface of lens is aspheric to decrease the spherical aberration. An anti-reflective coating (ARC) in the wavelength region of 8–12 μm is made on the lens to improve the transmittance.

The f-number of lens is determined as f/1.0 owing to the concern of noise equivalent temperature difference (NETD) of TIM. For a thermal imaging system, NETD is proportional to the square of the f-number [6] and is expected to be small enough for a good performance. The smaller f-number can significantly decrease NETD, but it may decrease depth of field (DOF) in the mean time, because DOF is also proportional to the f-number [7]. Obviously, there are tradeoffs between temperature sensitivity and DOF. Obviously, there are tradeoffs between temperature sensitivity and DOF. Fortunately, in most cases, the MTIS is used to obtain the temperature distribution of a focused target, therefore the effect of foreground and background blur can be neglected. Accordingly, the f-number should be as small as possible to decrease NETD.

Furthermore, to make sure that TIM can satisfy the ordinary measurement, the field of view (FOV) of TIM is determined to be about 39 degrees. In this case, a 2-meter-tall target (such as a human body) can be well-observed 3 m from the sensor. Then, the focal length of TIM can be determined as 10 mm, owing to the dice size of sensor (about 7 mm × 7 mm). It is very important to determine the appropriate FOV for a mobile-phone-based imaging device.

### 3.3. IRAPP Development

**Figure 4:**
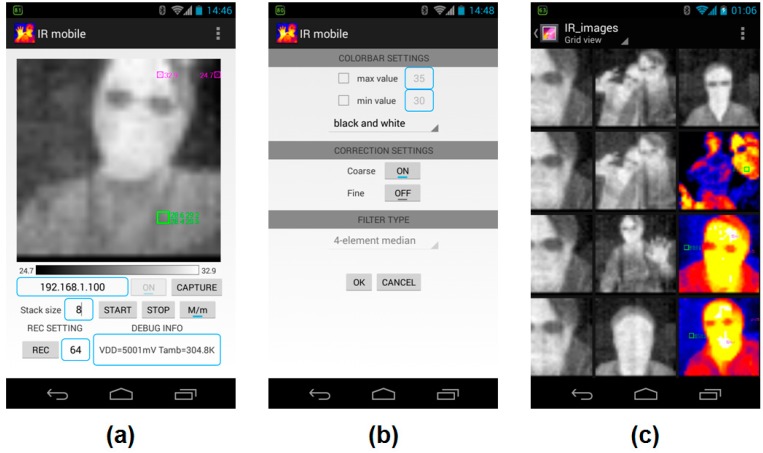
The graphic user interface of IR imaging application software (IRAPP). (**a**) The main page; (**b**) the settings page; and (**c**) the captured images stored in the micro SD card.

The IRAPP is developed with Android Software Development Kit (SDK) and the established graphical user interface (GUI) is shown in Figure 4. In the main page (Figure 4a), users can manually set the integration time to raise the signal-to-noise ratio (SNR). When running IRAPP, users can touch the main screen to determine the region of interest (ROI), and switch on the “M/m” button to monitor the extreme temperature area in real time. The color bar below the main screen indicates the temperature scale. In the settings page (Figure 4b), users can set the color bar pattern, correction algorithm, and filter type for the correction. All the captured images and data are stored in the micro SD card (Figure 4c).

**Figure 5:**
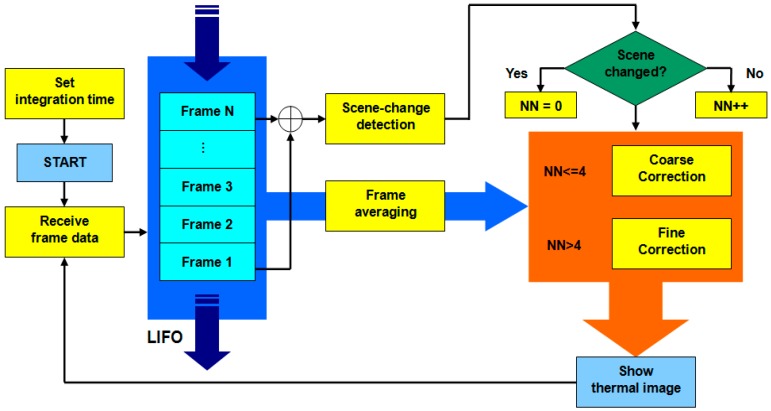
The IRAPP diagram flowchart.

The IRAPP diagram flowchart is shown in Figure 5. Considering that the time constant of detector may limit the steaming fluency, the frame averaging should be repeated whenever one frame comes in the stack, rather than a certain number of frames. Therefore, we use last-in-first-out (LIFO) method in the averaging algorithm.

In general, a non-uniformity correction (NUC) is required to perform good thermal imaging as a result of the inevitable hardware issue, such as salt-and-pepper noise, the irregular response of thermal detector, and inherent voltage difference among all pixels. For each pixel, a two-point linear correction is adopted to regularize the relation curves of raw image data against the temperature. The correction equation can be expressed as followed:

(1) $$y_{ij}=G_{ij}x_{ij}+O_{ij}$$

Here, *x_ij_* and *y_ij_* are the raw and corrected image data at position (*i*, *j*) respectively; *G_ij_* and *O_ij_* are the correction coefficients of gain and offset, respectively.

Furthermore, the coefficients of Equation (1), *i.e.*, *G_ij_* and *O_ij_*, should be updated with time to avoid any correction mistake when the behavior of hardware is changed by human-induced or unknown reason. Therefore, a self-adapted algorithm based on the artificial neural networks (ANN) is also adopted. Previous research has revealed the related principles, and the iteration equations can be obtained as follows: [8]

(2) $$G_{ij,\;n+1}=G_{ij,\;n}-2\alpha \;x_{ij}\;\left(y_{ij}-f_{ij}\right)$$

(3) $$O_{ij,\;n+1}=O_{ij,\;n}-2\alpha \;\left(y_{ij}-f_{ij}\right)$$

Here, *f_ij_* is the desired image data which is determined in advance; *n* the frame number; *α* the step-size parameter, which should be chosen to be small enough to ensure the stability of the iteration. The domain of *α* can be derived from Equations (2) and (3) that

(4) $$\frac{1}{{x_{ij}}^{2}+1}>\alpha >0$$

In general, the ANN algorithm usually takes thousands of frames for the recurrent calculation and thus lacks efficiency. Besides, once the observed scene has changed, the convergence rate of the ANN algorithm will thus vary. Such issue can be quite critical to the energy saving of mobile phone.

To maximize the efficiency and decrease the power consumption of APP computing, we propose two kinds of scenario for the NUC: “Coarse Correction” and “Fine Correction”. A scene-change detection algorithm is implemented to distinguish between these two kinds of scenario, as shown in Figure 5. By detecting the changing of pixel position of maximum temperature value between the first and last frames in the stack, it is simple to recognize whether the scene has changed or not. When the scene keeps changing, IRAPP use two-point linear correction, as in the case of Coarse Correction; when the scene ass not changed over 4 frames, IRAPP starts using the ANN algorithm, as in the case of Fine Correction.

## 4. Experimental Results

### 4.1. Performance Overview

**Figure 6:**
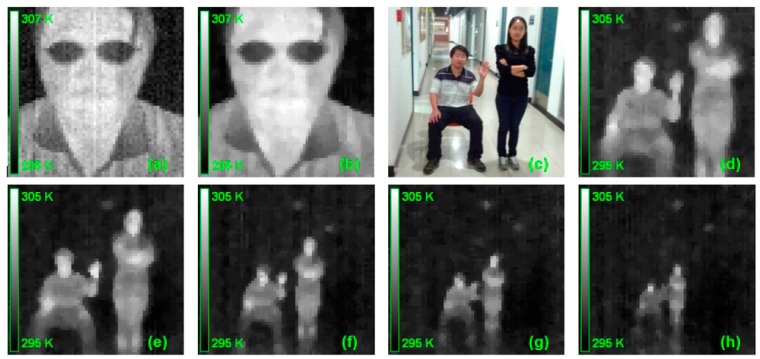
The performance of MTIS. (**a**) The raw thermal image of human face; (**b**) the corrected result of (**a**); (**c**) two people in different poses; and (**d**–**h**) the thermal image of (c) at 2, 3, 4, 5, and 6 m from the MTIS.

The performance for the current MTIS is demonstrated in Figure 6. Clearly, one can see that the original imaging result of TIM is usually non-uniform and full of background noise (Figure 6a). After utilizing the correction algorithm, the imaging quality is significantly improved to recognize the whole facial feature (Figure 6b).

We also tested the imaging quality of MTIS at different observation distances. Here, two people in sitting and standing positions (Figure 6c), which were to indicate two objects with different profiles, can be well recognized, even at 6 m from the MTIS (Figure 6d–h). This shows the practical value of the current mobile system.

**Figure 7:**
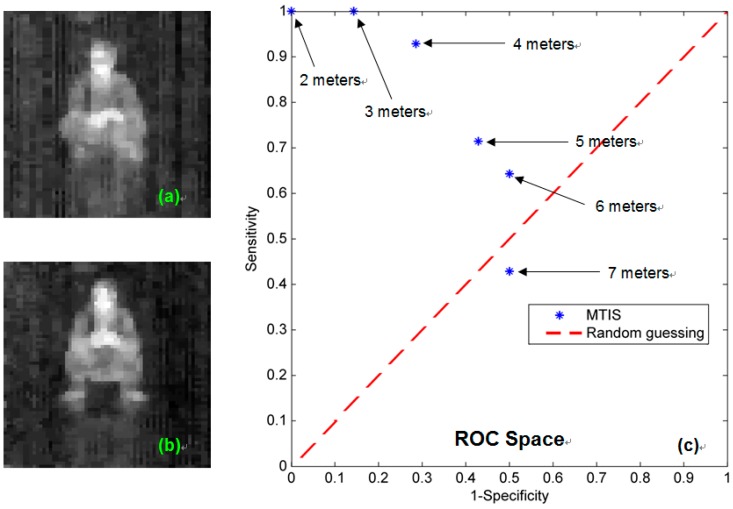
The recognition experiments of MTIS. (**a**) Sample pattern of the sitting position; (**b**) sample pattern of the crouched position; and (**c**) the receiver operating characteristic (ROC) space plot.

In order to further evaluate the performance of MTIS, we additionally performed tests on several groups of thermal pattern of the same persons by using MTIS. Each group indicates the thermal imaging results at different distances from the MTIS, and contains two kinds of body position patterns, sitting and crouched, as shown in Figure 7a,b, respectively. Then, we solicited 14 volunteers for a recognition experiment. After watching the pattern, each volunteer individually answers the question as which kind of body position is in the thermal image.

Based on the experiment results, we made a sensitivity and specificity analysis of pattern recognizing ability of MTIS, as listed in Table 1 and shown in Figure 7c. Here, the sitting position is labeled as positive, and the crouched position is labeled as negative. We can see that when the observation distance of MTIS is within 6 m, the present measurement can guarantee that the detected result will be better than random guessing.

**Table 1:** Sensitivity and specificity analysis of pattern recognizing ability of MTIS.

Distance	Sensitivity	Specificity	Accuracy
2 m	1.000	1.000	1.000
3 m	1.000	0.857	0.929
4 m	0.929	0.714	0.821
5 m	0.714	0.571	0.643
6 m	0.643	0.500	0.571
7 m	0.429	0.500	0.464

In the quantitative analysis of MTIS, we follow a series of standard process to quantify the thermal sensitivity and available detective distance of MTIS. In general, there are two representative parameters to measure: NETD and the minimum resolvable temperature difference (MRTD).

### 4.2. NETD Behavior

NETD represents the temperature value corresponding to the noise signal. In the practical examination, it is defined as the standard deviation of image data within a certain range of temperature. We placed the TIM in front of the black body furnace and made sure that the vision of the sensor is covered by the cavity aperture of the furnace. In the IRAPP, the integration time was set as 8, 16, 32, and 64 frames. Then, we follow the American Society for Testing and Materials (ASTM) standard E1543 to measure and calculate the NETD behavior as listed in Table 2. It indicates that the NETD decreases with the increase of the integration time. For commercial thermal imagers, the typical range of NETD is about 50–200 mK. Therefore, when the MTIS is applied to a situation that requires precise nondestructive measurement, an integration time longer than 16 frames is reliable.

**Table 2:** NETD behavior of TIM.

Integration Time	NETD (mK)
8 frames	302.52
16 frames	144.19
32 frames	99.41
64 frames	70.25

### 4.3. MRTD Behavior

MRTD is another important measure for assessing the performance of the thermal imaging system. It represents the system’s temperature sensitivity and the capacity to distinguish details of the measured target. In the practical examination, we used several pieces of standard 4-bar pattern as the measured target [9]. We place the 4-bar pattern between the black body furnace and the TIM, and then follow the ASTM standard E1213 to measure MRTD corresponding to spatial frequency. The pattern’s spatial frequency is defined as follows:

(5) $$F=\frac{10^{-3}D}{s}=\frac{10^{-3}D\cdot l}{h}$$

Here, *F* is the spatial frequency (cycles/mrad); *D* the observation distance from target to TIM (m); *s* the distance between two center lines of bars in the horizontal direction (m); *l* the number of line pair; and *h* the height of pattern (m).

**Figure 8:**
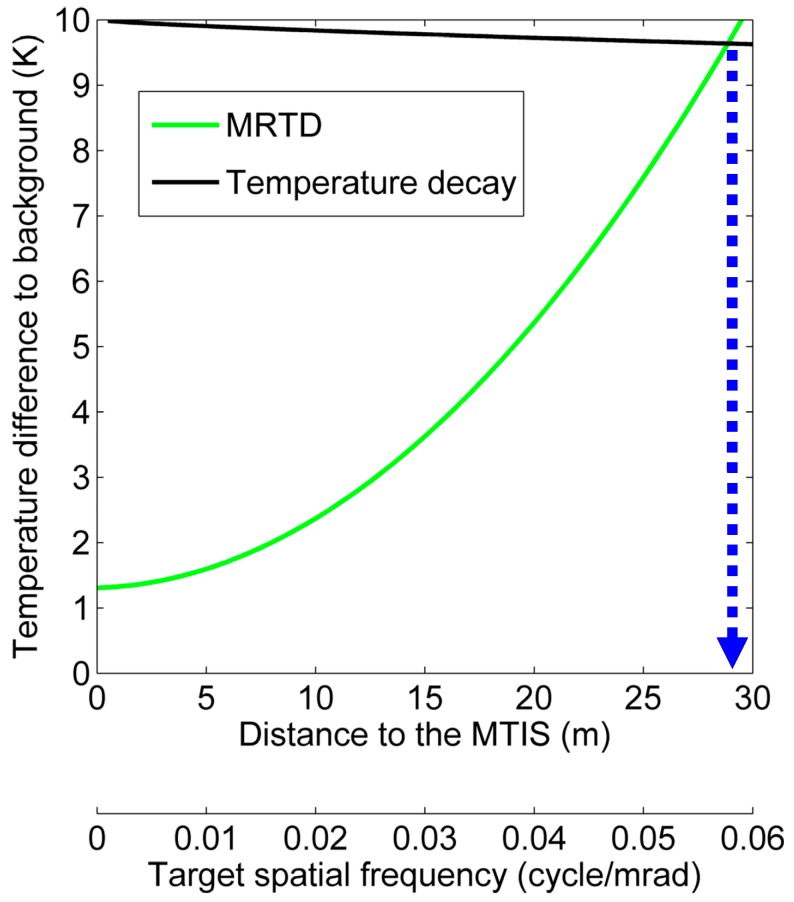
The intersection point of two curves indicates the estimation for the maximum working distance of MTIS. The black curve is derived by LOWTRAN (Low Resolution Atmospheric Radiance and Transmittance Model), which is a propagation model developed by U.S. Air Force Geophysics Laboratory (AFGL) for predicting atmospheric transmittance and background radiance.

Based on the MRTD behavior, the available detective distance of MTIS can subsequently be estimated. Here, we assume a scenario that MTIS is detecting the existence of human body at room temperature. The target is about 2 m, and the temperature difference between target and background is about 10°C. According to the Johnson criteria, for a 50% probability of “recognition,” the minimum required resolution of target should be at least 4 line pairs [10]. As shown in Figure 8, the green curve indicates MRTD behavior of MTIS; the black curve indicates the temperature decay of the target. Then, the maximum working distance can be determined by the intersection point of two curves [11]. The result indicates that the maximum detective distance of MTIS is about 29 m.

## 5. Discussion

At this stage, the practicability of MTIS has been successfully demonstrated. Under the conditions of cost-efficiency, the performance of MTIS could well meet anticipative requirement. Compared with the existing low-cost thermal imagers, we make a tabular form of comparison as listed in Table 3. Apparently, MTIS has significant advantage in terms of resolution, size, price, *etc.*

As has been mentioned in Section 2.2 of this article, the price of a microbolometer is much higher than that of a thermopile, and thus such issue reflects a considerable difference between FLIR E4 and the other thermal imagers in the end price. In the past, the spatial resolution of a thermopile was low due to the limitation of wafer fabrication technology at that time. Therefore, the thermopile array cannot compete in resolution with the microbolometer. Today, rapid advances in thermopile technology have significantly improved the overall performance of the thermopile device at an even lower cost. This guarantees that MTIS can have higher resolution than HIOKI 3460 and IRI 1011, and run the lowest price among these four thermal imagers.

Moreover, because of the combination of thermal imaging module and mobile phone, the module itself need no additional computing and display unit, and thus size and cost of MTIS can be evidently reduced. In fact, as the computing capability of mobile phone is still increasing with the change of generation, the hardware of MTIS can be updated easily in the coming time. 

**Table 3:** Comparison among four low-cost thermal imagers.

	MTIS	HIOKI 3460	IRI 1011	FLIR E4
Detector	Thermopile	Thermopile	Thermopile	Microbolometer
Resolution	64 × 62	8 × 8	16 × 16	80 × 60
Range (°C)	−20 to 300	−50 to 1000	−10 to 300	−20 to 250
NETD (mK)	300	N/A	300	150
Dimension (mm)	36 × 36 × 44	165 × 55 × 123	120 × 125 × 80	244 × 95 × 140
Weight (g)	<200	700	600	575
Price (USD)	<500 *	700–900 **	800–1000 **	1200–1800 **

* Our expecting price. As far as we are informed, the price of thermopile array chip is about 500 US dollars for over 50 pieces, and the price of germanium lens is about 100 US dollars. The cost of MTIS can be further decrease in mass production; ** Depends on purpose and accessory.

**Figure 9:**
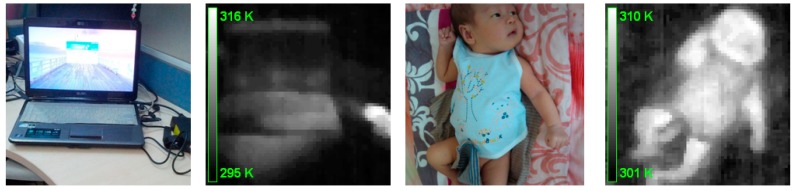
Thermal image captured by MTIS. (**a**) a laptop with a hot AC adapter; (**b**) the thermal image of (a); (**c**) a two-month-old infant; and (**d**) the thermal image of (c).

At present, we have applied MTIS to several family-related applications, such as thermal detection in the surrounding area (Figure 9a,b) and infant temperature monitoring (Figure 9c,d). Instead of traditional temperature measurement, previous research revealed that forehead measurement for newborns could also be reliable and effective [12]. Since the core of the current work is to develop the hardware and software system for MTIS, additional applications will not be addressed here for brevity.

In the near future, along this direction, one could propose a series of novel medical applications for infant health care based on the MTIS. For example, sleeping in the prone position is regarded as a significant risk factor for sudden infant death syndrome (SIDS) [13], because the heat loss from head is a considerable ratio of body heat dissipation [14] and the thermoregulation mechanism of infants is immature [15]. Therefore, for a SIDS prevention system, it should be able to monitor not only the body temperature, but also the sleeping posture of infants. Clearly, the MTIS would work well to fulfill this entirely safe task. By using MTIS to monitor the thermal image of the infant face, parents can take care of their babies anytime and anywhere with the IRAPP on mobile phone, and receive instant alerts when abnormal temperature or an inappropriate position of the infant face is detected. Because of the body proportions of infants, the pattern recognition for infant face can be achieved even in low spatial resolution.

## 6. Conclusions

Overall, the present achievements of MTIS project promise a new solution for low-cost thermal imager with accessibility, reliability, and cost-efficiency. On the foundation of a mobile phone, the MTIS can be expected to be more pervasively important than the traditional thermal imager in various nondestructive measurements. In the near future, we plan to investigate more potential applications in daily life.
